# A Case of Monstrous Birth

**Published:** 1793

**Authors:** Alexander Mather

**Affiliations:** Surgeon at York.


### VII. *A Caſe of monſtrous Birth. By the ſame.*

HISTORIES of monſtrous births ſeem to become intereſting in proportion as they add to the varieties in this way already deſcribed, or as they tend to afford uſeful hints to the practitioner of midwifery in ſimilar caſes. Conſidered in the latter point of view, the following caſe cafe may, perhaps, be deemed worthy of being recorded.

In the evening of Auguft 16th, 1789, I was called to the wife of a miller in this town who had gone her full time. She informed me that fhe had had no pain, but that having felt an inclination to make water, fhe had got up, and immediately felt fomething drop into the vagina. On examining her I found the foot of a child without the os externum; another foot in the imperfectly dilated os uteri; and a third limb prefenting itfelf a little higher up. Being unwilling to occafion unneceffary pain to the patient, I did not attempt to afcertain what this laft was, but conjecturing that it was a hand that had flipped into the pelvis, and that it would not impede the progrefs of the labour as a foot prefentation, I waited patiently for the coming on of the pains. In a fhort time they came on, and on examining during a pain, I was furprized to find that the higheft limb feemed to defcend in an equal proportion with the reft. I now thought it proper to afcertain whether it was a hand, and by the help of flow and weak pains, was foon convinced that it was a third foot. As the circumftances of the cafe were uncommon, and I had reafon to think there was fomething preternatural ternatural in the form of the fœtus, I requeſted the aſſiſtance of a gentleman who had had conſiderable practice in midwifery. He was ſoon convinced that three feet preſented; and ſuppoſing that two children had ſlipped together into the pelvis, adviſed me to advance the two firſt feet and retard the other as much as poſſible, leſt, by deſcending together, the bodies might be ſo compreſſed in the pelvis as to make the delivery very difficult. This I accordingly, by gentle means, attempted to do, but was more and more convinced of a preternatural formation of the fœtus, the three limbs deſcending ſtill together: I therefore ſoon deſiſted, wiſhing to leave the caſe to nature; but the gentleman I had called in ſtill maintaining his opinion, made likewiſe an attempt to return the higheſt limb, and for ſome time uſed conſiderable force for that purpoſe, but without effect. During his efforts a fourth foot came within reach; and we now agreed to leave the caſe to nature.

In order to watch the progreſs of ſo curious a labour I continued at the bed ſide, and the pains increaſing, I plainly perceived the fourth foot deſcend with the others, and when it reached the os externum I drew it down to the ſame length, and paſſing my hand between the two pair pair of legs, I felt the junction of two bodies at the navel; I therefore enclofed the four limbs in a cloth, and was pleafed to find the fœtus defcend eafily with every pain.

Fearing fome obftruction at the fhoulders, (if this mal-conformation of parts fhould extend to them,) as foon as the bodies were advanced to the ribs I fought for the arms and fucceffively brought down four. I then immediately paffed my finger above the brim of the pelvis and foon found a mouth, by which I brought down a head into the cavity of the pelvis; it paffed the os externum very eafily, and was immediately followed by another head presenting to us two male children completely and naturally formed in every part, except that at the top of the fternum a junction of their bodies began, which was continued down to the navel. They were fupported in the uterus by one umbilical cord which was attached to one large placenta. The cord terminated exactly in the center of the angle made by the junction of the bellies of the two children.

Being defirous of retaining intire fo extraordinary a fœtus, I did not examine the ftructure of any of the internal parts; but it is evident that there is only one fternum common to both, and and that the ribs riſe by one origin from it, and then divaricate to form a ſeparate thorax to each child. The children were each as large as many are from ſingle births of nine months, but not ſo large ſingly as the former children of the ſame woman had been.

*York,*

*September* 8*th*, 1792.

